# Design of New Polyaspartamide Copolymers for siRNA Delivery in Antiasthmatic Therapy

**DOI:** 10.3390/pharmaceutics12020089

**Published:** 2020-01-22

**Authors:** Emanuela Fabiola Craparo, Salvatore Emanuele Drago, Nicolò Mauro, Gaetano Giammona, Gennara Cavallaro

**Affiliations:** 1 Lab of Biocompatible Polymers, Department of Biological, Chemical and Pharmaceutical Sciences and Technologies (STEBICEF), University of Palermo, via Archirafi 32, 90123 Palermo, Italy; emanuela.craparo@unipa.it (E.F.C.); salvatoreemanuele.drago@unipa.it (S.E.D.); nicolo.mauro@unipa.it (N.M.); gaetano.giammona@unipa.it (G.G.); 2 Fondazione Umberto Veronesi, Piazza Velasca 5, 20122 Milano, Italy

**Keywords:** siRNA, STAT6, polyaspartamide, pegylation, polyamine, polyplexes, asthma

## Abstract

Here, a novel protonable copolymer was realized for the production of polyplexes with a siRNA (inhibitor of STAT6 expression in asthma), with the aim of a pulmonary administration. The polycation was synthesized by derivatization of α,β-poly(N-2-hydroxyethyl)d,l-aspartamide (PHEA) with 1,2-Bis(3-aminopropylamino)ethane (bAPAE) in proper conditions to obtain a PHEA*-g-*bAPAE graft copolymer with a derivatization degree in amine (DD_bAPAE_%) equal to 35 mol%. The copolymer showed a proper buffering behavior, i.e., ranging between pH 5 and 7.4, to potentially give the endosomal escape of the obtained polycations. In effect, an in vitro experiment demonstrated the effect on biological membranes of the copolymer on bronchial epithelial cells (16-HBE) strongly dependent on the pH of the medium, i.e., higher at pH 5. bAPAE-based copolymers were further obtained with an increasing pegylation degree, i.e., equal to 1.9, 2.7, and 4.4 mol%, respectively. All the obtained copolymers were able to complex siRNA at a N/P ratio that decreases as the pegylation degree increases. At the same time, the tendency of polyplexes to aggregate and the capability to interact with mucin also decreases as the pegylation in the copolymer increases. Gene silencing experiments on 16-HBE showed that these copolymers have a significant role in improving the intracellular transport of naked siRNA, where the presence of PEG does not seem to hinder the cellular uptake of polyplexes. The latter obtained at polymer/siRNA weight ratio (R) equal to 10 with PHEA*-g-*PEG(C)*-g-*bAPAE also seems to be not susceptible to the presence of mucin, avoiding the polyanionic exchange of complexed siRNA, thus showing adequate behavior to be used as an effective vector for siRNA.

## 1. Introduction

**Figure 1:**
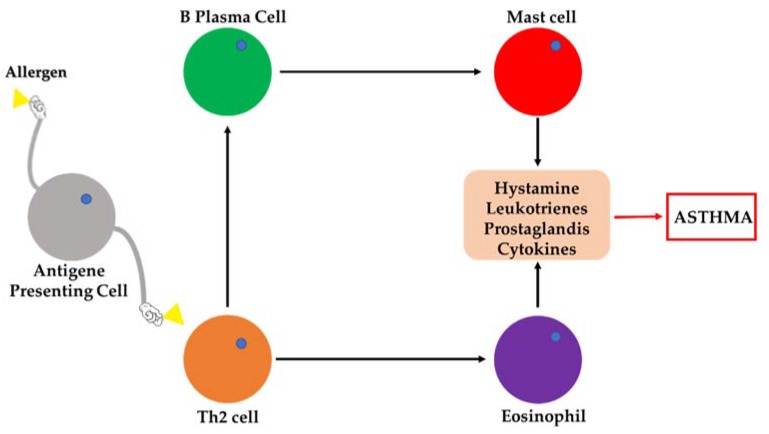
Schematic representation of the inflammatory cascade in allergic asthma.

Although it is not a deadly disease, asthma can be highly debilitating, resulting in irreversible lung damage [1]. The international guidelines of the Global Initiative for Asthma (GINA) define asthma as a pathological state characterized by chronic inflammation of the airways and reversible limitation of air flow. However, persistent lung inflammation could cause airway obstruction or hyper-reactivity, so that when the treatment is inadequate, airway re-modelling can occur and the obstruction becomes irreversible [1]. The inflammatory cascade in allergic asthma, which involves the lung infiltration of eosinophils, T lymphocytes, mast cells, and other inflammatory cells, is schematically summarized in Figure 1.

The conventional drug therapy includes both bronchodilators (such as β-2 adrenergic agonists and anticholinergics) to control symptoms, and corticosteroids to reduce the inflammatory process. Inhaled corticosteroids represent an effective first-line treatment in mild asthma, but they have therapeutic limitations in patients with moderate to severe asthma due to several side effects, although the local administration allows to significantly reduce the administered dose [2,3].

The conventional therapies, therefore, are often associated with a series of limitations that have pushed the research towards the identification of new biological targets for treatment of the pathology. It has been seen that in individuals suffering from asthma there is overexpression of numerous genes and proteins, such as Signal Transducer and Activator of Transcription 6 (STAT6) [4], Plasminogen Activator Inhibitor-1 (PAI-1) [5], and Spleen Tyrosine Kinase (Syk) [6]. In particular, STAT6 regulates the T helper type 2 (Th_2_) immune response, PAI-1 is associated with asthma severity because of its role in airway remodeling, while Syk is implicated as central immune modulator promoting allergic airway inflammation; thus, their inhibition could be an effective therapeutic approach in asthma.

For these reasons, an alternative therapeutic approach of pathological states caused by an increase in the expression of some genes, as in the case of asthma, could be gene therapy, through the administration of small interference RNA (siRNA). These consists of small double-stranded RNA fragments capable of triggering the degradation of a specific mRNA [7] and therefore ultimately capable of blocking the synthesis of certain proteins. Recently, the true efficacy of siRNA directed against particular targets, such as STAT6, PAI-1, and Syk for the treatment of asthma was demonstrated through appropriate in vitro and in vivo studies [4,5,6].

However, despite the potential of this genetic material, it is well known that it cannot be administered as it is, also if given directly in situ, but requires the use of particular vectors capable of conveying it into the body [7,8]. The use of siRNA as therapeutic agents necessarily requires the use of a vector, which by neutralizing the negative charge may allow them to enter cells, as well as increasing their stability against enzymatic degradation by nucleases. Among gene vectors, polymeric materials have many advantages, as they can carry large quantities of genetic material and can be chemically derivatized to obtain systems specifically oriented towards particular target tissues [9,10]. The cationic character of such polymers, necessary for the establishment of interactions with negative-charged gene material to form polyplexes, is conferred by protonable amino groups at physiological or neutral pH. However, other peculiarities are often required of the polymeric material, therefore a starting material easily modifiable by simple chemical reactions is highly sought after.

Considering the above, the aim of the present experimental work was to realize a novel protonable polymeric derivative able to form a stable electrostatic complex with the chosen siRNA, and to delivery it, through the inhalation route, at the bronchial level for the realization of an innovative formulation for the management of asthma. We have chosen to work with the α,β-poly-N-2-hydroxyethyl-dl-aspartamide (PHEA) as the starting polymeric material [11], being a highly soluble in water, biocompatible, non-immunogenic, non-antigenic polymer, already used for the development of highly performing polymeric gene vectors [12,13], as well as many other drug carriers [14,15,16,17]. Moreover, as molecule to conjugate to the PHEA backbone, in order to give the cationic behavior, we have chosen an oligoamine, the 1,2-Bis(3-aminopropylamino)ethane (bAPAE), that could give a good complexing capability, an improvement of cell internalization, associated with a good cytocompatibility [18]. As genetic material has been chosen a therapeutic siRNA able to reduce the expression of STAT6, that is one of the most important transcription factors that regulate the production of Th2 cytokines and effector functions mediated by Th2 cytokines [19,20,21,22], which seems to have a major role in the mechanism that initiates an asthmatic attack.

In addition to complexing ability to the genetic material, the copolymer forming the siRNA complex should possess additional characteristics, after administration by the pulmonary route, to further diffuse trough the mucus layer present at the airway level, until reach the bronchial epithelial cells. The latter are the main target for the siRNA delivery. To confer penetrating mucus capacity to the system it is possible to increase the superficial hydrophilicity of the system, thus reducing interactions with the protein chains of the mucin; for this reason, the PHEA backbone was also conjugated with a proper amount of poly(ethyleneglycole) (PEG) [23,24]. Therefore the potential of this new polyaspartamide copolymers as material able to complex and delivery a specific siRNA for antiasthmatic therapy was tested.

## 2. Materials and Methods

### 2.1. Materials

Triethylamine (TEA), Bis(4-nitrophenyl)carbonate (BNPC), anhydrous *N,N’*-dimethylformamide (a-DMF), 1,2-Bis(3-aminopropylamino)ethane (bAPAE), *O*-(2-Aminoethyl)-*O’*-methyl poly(ethylene glycol) 2000 (H_2_N-PEG_2000_) (0.4 mmol NH_2_/g), disuccinimidylcarbonate (DSC), dichloromethane, aceton, diethylether, 2,4,6-Trinitrobenzenesulfonic acid (TNBS), agarose, ethidium bromide, mucine from porcin stomach were purchased from Sigma-Aldrich (Milan, Italy). All used reagents were of analytic grade.

Duplexed siRNA were purchased from Biomers.net (Ulm, Germany). The gene target sequences (5′→3′) are: CAGUUCCGCCACUUGCCAA (sense), UUGGCAAUGGCGGAACUG (antisense).

α,β-poly(N-2-hydroxyethyl)-d,l-aspartamide (PHEA) was synthetized via polysuccinimide (PSI) reaction with ethanolamine in DMF solution, and purified according to a previously reported procedure [11]. 

^1^H-NMR (300 MHz, D_2_O, 25°C, TMS): δ 2.71 (m, 2H_PHEA_, –COCHC**H_2_**CONH–), δ 3.24 (m, 2H_PHEA_, –NHC**H_2_**CH_2_O–), δ 3.55 (m, 2H_PHEA_, –NHCH_2_C**H_2_**OH), δ 4.59 [m, 1H_PHEA_, –NHC**H**(CO)CH_2_–].

### 2.2. Copolymer Synthesis

#### 2.2.1. General Procedure for the Derivatization and Characterization of α,β-poly(*N*-2-hydroxyethyl)d,l-aspartamide with 1,2-Bis(3-aminopropylamino)ethane (PHEA*-g-*bAPAE)

Derivatization of PHEA with 1,2-Bis(3-aminopropylamino)ethane (bAPAE) was carried out by using Bis(4-nitrophenyl) carbonate (BNPC) as coupling agent. Two hundred milligrams of PHEA (1.26 mmol of repeating units (RU)) were dissolved in 4 mL of a-DMF; after complete solubilization, 230 mg of solid BNPC was added. The solution was stirred at 40 °C for 4 h. Simultaneously, 922.71 μL of bAPAE was dissolved in 7 mL of a-DMF. After activation time, the resulting polymeric solution was added dropwise and slowly to bAPAE solution. The reaction was carried out under and continuous stirring at 25 °C for 20 h. The amounts of each reagent were properly determined accordingly to R_1_ = (mmol of BNPC/mmol of functionalizable RU on PHEA) = 0.6 and R_2_ = (mmol of bAPAE/mmol of functionalizable RU on PHEA) = 4.

After this time, the polymer was isolated from reaction mixture by precipitation in mixture 2:1 *v/v* diethyl ether/dichloromethane and the supernatant was removed by centrifugation at 4 °C for 8 min, at 9800 rpm. The obtained solid product was washed with acetone, until the pH of a mixture between the washing acetone with water (vol:vol 1:1) was neutral. Then, the obtained product was dried under vacuum. The solid residue was dissolved in double distilled water and then the solution was purified by dialysis (SpectraPor Dialysis Tubing, at MWCO 25 kDa), for two days against basic water (NaOH) and for other three days against bidistilled water, subsequently the solution was freeze-dried and stored for further characterization. PHEA*-g-*bAPAE graft copolymer was obtained with a yield of 80 wt % based on the starting PHEA.

^1^H-NMR (300 MHz, D_2_O pD 5, 25 °C, TMS): δ 1.70–2.20 (m, 4H_bAPAE_, –NHCH_2_CH_2_CH_2_NHCH_2_CH_2_NHCH_2_CH_2_CH_2_NH–), δ 2.73 (m, 2H_PHEA_, –COCHCH_2_CONH–), δ 3,12 (m, 8H_bAPAE_, –NHCH_2_CH_2_CH_2_NHCH_2_CH_2_NHCH_2_CH_2_CH_2_NH_2_), δ 3.23 (m, 2H_PHEA_, –NHCH_2_CH_2_O–), 3,38 (m, 4H_bAPAE_, –CONHCH_2_CH_2_–, –CH_2_CH_2_CH_2_NH_2_), δ3.54 (m, 2H_PHEA_, –NHCH_2_CH_2_OH), δ 3.60 (m, 4H_PEG_, –[OCH_2_CH_2_O]_44_–), δ 4.02 (m, 2H_PHEA_, –NHCH_2_CH_2_OCO–), δ 4.62 (m, 1H_PHEA_, –NHCH(CO)CH_2_–). The content of amine was also determined by TNBS assay [12].

#### 2.2.2. General Procedure for the Derivatization and Characterization of PHEA with methoxy polyethylene glycol amine (H_3_CO-PEG-NH_2_)

Derivatization of PHEA with different amount of H_3_CO-PEG-NH_2_, was carried out by using N,N’-disuccinimidyl carbonate (DSC) as coupling agent [23]. Five hundred milligrams of PHEA (6.32 mmol of RU) was dissolved in 10 mL of a-DMF at 40 °C and then a proper amount of triethylamine (TEA), as catalyst, and DSC were added; subsequently, the reaction mixture was left at 40 °C for 4 h.

After the activation time, the latter dispersion of DSC-activated PHEA was added drop-wise to increasing volumes of H_3_CO-PEG-NH_2_ dispersions in a-DMF, at a concentration of 50 mg/mL. Then, the obtained mixture reactions were left at 25 °C for 18 h. The amounts of TEA, DSC and PEG were added according to the following moles ratios, as reported in Table 1.

After this time, each polymer was isolated from reaction mixture by precipitation diethyl ether and the supernatant was removed by centrifugation at 4 °C for 8 min, at 9800 rpm. The obtained solid product was washed with acetone one time and then, the obtained product was dried under vacuum. The solid residue was dissolved in double distilled water and then the solution was purified by dialysis (SpectraPor Dialysis Tubing, at MWCO 25 kDa), subsequently freeze-dried and stored for further characterization. PHEA*-g-*PEG graft copolymers were obtained with a yield of 220 wt % based on the starting PHEA. Three different PHEA*-g-*PEG graft copolymers in terms of PEG grafted on the PHEA backbone, that were named PHEA*-g-*PEG(A) and PHEA*-g-*PEG(B) and PHEA*-g-*PEG(C).

^1^H-NMR (300 MHz, D_2_O, 25 °C, TMS): δ 2.71 (m, 2H_PHEA_, –COCHCH_2_CONH–), δ 3.24 (m, 2H_PHEA_, –NHCH_2_CH_2_O–), δ3.55 (m, 2H_PHEA_, –NHCH_2_CH_2_OH), δ 3.60 (m, 4H_PEG_, –[OCH_2_CH_2_O]_44_–), δ 4.59 (m, 1H_PHEA_, –NHCH(CO)CH_2_–).

#### 2.2.3. General Procedure for the Derivatization and Characterization of PHEA*-g-*PEG with bAPAE

Derivatization of PHEA*-g-*PEG with bAPAE was carried out by using BNPC as coupling agent. 232 mg of PHEA*-g-*PEG(A), 278 mg of PHEA*-g-*PEG(B), or 329 mg of PHEA*-g-*PEG(C) (corresponding to 1.26 mmol of functionalizable RU) was dissolved in 4 mL of a-DMF; after complete solubilization, 230 mg of solid BNPC was added. The solution was stirred at 40 °C for 4 h. Simultaneously, 922.71 μL of bAPAE was dissolved in 7 mL of a-DMF. The reagents were added accordingly to R_1_ = (mmol of BNPC/mmol of functionalizable RU on PHEAPEG) = 0.6 and R_7_ = (mmol of bAPAE/mmol of functionalizable RU on PHEAPEG) = 4.

After activation time, the resulting polymeric solution was added dropwise and slowly to bAPAE solution. The reaction was carried out under continuous stirring at 25 °C for 20 h. After this time, the polymer was isolated from reaction mixture by precipitation in mixture 2:1 *v/v* diethyl ether/dichloromethane and the supernatant was removed by centrifugation at 4 °C for 8 min, at 9800 rpm. The obtained solid product was washed with acetone, until the pH of the washing surnatant was neutral. Then, the obtained product was dried under vacuum. The solid residue was dissolved in double distilled water and then the solution was purified by dialysis (SpectraPor Dialysis Tubing, at MWCO 25 kDa), subsequently the solution was freeze-dried and stored for further characterization. PHEA*-g-*PEG*-g-*bAPAE graft copolymers were obtained with a yield of 80 wt% based on the starting PHEA*-g-*PEG.

^1^H-NMR (300 MHz, D_2_O pD 5, 25 °C, TMS): δ 1.70–2.20 (m, 4H_bAPAE_, –NHCH_2_CH_2_CH_2_NHCH_2_CH_2_NHCH_2_CH_2_CH_2_NH–), δ 2.73 (m, 2H_PHEA_, –COCHCH_2_CONH–), δ 3.12 (m, 8H_bAPAE_, –NHCH_2_CH_2_CH_2_NHCH_2_CH_2_NHCH_2_CH_2_CH_2_NH_2_), δ 3.23 (m, 2H_PHEA_, –NHCH_2_CH_2_O–), 3,38 (m, 4H_bAPAE_, –CONHCH_2_CH_2_–, –CH_2_CH_2_CH_2_NH_2_), δ3.54 (m, 2H_PHEA_, –NHCH_2_CH_2_OH), δ 3.60 (m, 4H_PEG_, –[OCH_2_CH_2_O]_44_–), δ 4.02 (m, 2H_PHEA_, –NHCH_2_CH_2_OCO–), δ 4.62 (m, 1H_PHEA_, –NHCH(CO)CH_2_–).

### 2.3. Determination of the Amine Content

The content of amine-terminated side chains was also determined by TNBS assay. A stock solution of PHEA*-g-*bAPAE or PHEA*-g-*PEG(A)*-g-*bAPAE or PHEA*-g-*PEG(B)*-g-*bAPAE or PHEA*-g-*PEG(C)*-g-*bAPAE (5 mg/mL) was prepared in a borate buffer (0.1M Na_2_B_4_O_7_·H_2_O, pH 9.3). An aliquot of this solution (50 μL) was added to a cuvette containing 900 μL of borate buffer and 50 μL of 0.03 M TNBSA solution. After 120 min incubation, absorbance at λ 500 nm was measured and compared with that estimated for the reaction of H_2_N-PEG-OCH_3_ (–NH_2_ in the range between 0.01 and 0.001 mmol/mL) with TNBSA.

### 2.4. Size Exclusion Chromatography

Weight-average molecular weight (${\bar{M}}_{w}$), polydispersity index (${\bar{M}}_{w}/{\bar{M}}_{n})$, of each copolymer was determined by a size exclusion chromatography (SEC) analysis, performed using Tosho Bioscience TSK-Gel G4000 PWXL and G3000 PWXL columns(Sursee, Switzerland) connected to an Agilent 1260 Infinity Multi-Detector GPC/SEC system(Santa Clara, United States), and a refractive index detector. Analyses were performed with buffer citrate/phosphate 0.15 M + 0.1 M NaCl pH 5 as eluent with a flow of 1 mL/min and poly(ethylene oxide) standard (40 kDa) to obtain the calibration curve. The column temperature was set at 30 °C.

### 2.5. Potentiometric Titration of PHEA-g-bAPAE Graft Copolymer

#### 2.5.1. Qualitative Titration of PHEA*-g-*bAPAE Copolymer

To determine the relative buffering capacity of PHEA*-g-*bAPAE copolymer, potentiometric acid−base titrations were performed. Typically, 6 mg of copolymer was dissolved in 30 mL in 0.1 N NaCl, used as ionic strength stabilizer, and the pH was adjusted to nearly 10.0 using 0.1 N sodium hydroxide. Then the mixture was titrated by gradually adding 20 μL of 0.1 N HCl until reaching pH 3. Titrations of comparable amounts of PHEA, and bAPAE, calculated considering the derivatization degree of the PHEA*-g-*bAPAE copolymer, at the same concentration present in PHEA-bAPAE, were also studied.

#### 2.5.2. Determination of the pKa Values of PHEA*-g-*bAPAE Copolymer by Potentiometric Titration

30 mg of PHEA*-g-*bAPAE were dissolved in 0.1 N degassed NaCl (30 mL), used as ionic strength stabilizer, and termostated at 25 °C under argon atmosphere. The solution was then titrated using 0.05 N HCl until pH 3 under inhert conditions. Backward titrations were performed using 0.05 N NaOH. For all titrations an AMEL 631 differential electrometer was used, which was calibrated against a set of multiple standard buffers (2.50 ± 0.01 ≤ pH ≤ 10.00 ± 0.01). The pKa values of the amine groups were extrapolated using the De Levie method of acid−base chemical equilibria for polyelectrolytes.

### 2.6. Biological Studies

#### 2.6.1. Cell Culture

In this study, an immortalized normal bronchial epithelial cell line (16-HBE) was used (furnished by Istituto Zoo-profilattico of Lombardia and Emilia Romagna). 16-HBE cells were maintained in a humidified atmosphere of 5% CO_2_ in air at 37 °C, cultured as adherent monolayers in Dulbecco’s Modified Eagle’s medium (DMEM) (EuroClone), supplemented with 10% fetal bovine serum (FBS) (Gibco), 2 mM l-glutamine (EuroClone), 100 U/mL penicillin, 100 g/mL streptomycin, and 0.6 g/mL amphotericin B (Sigma–Aldrich, Milan, Italy).

#### 2.6.2. Membrane Destabilization Study

16-HBE cells were plated on a 48-well plate at a cell density of 10.000 cells/well in DMEM containing 10% FBS. After 24 h of incubation, the medium was removed and then the cells were incubated with 100 µL of DPBS (pH 7.4) or 20 mM MES (pH 5.5, 130 mM NaCl) containing PHEA*-g-*bAPAE (0.5 mg/mL) for 20 min at 37 °C; in the same condition, blank analysis was also performed. After this time, 100 µL of Tripan Blue 0.2% was added to each well. After 1 min of incubation, the supernatant was removed and cells were observed with miscroscope (Axio Cam MRm, Zeiss, Oberkochen, Germany). All analysis was performed in triplicate. Simultaneously, 16-HBE cells were plated on a 24-well plate at a cell density of 100,000 cells/well in DMEM containing 10% FBS. After 24 h of incubation, the medium was removed and then the cells were incubated with 100 µL of PBS (pH 7.4) or 20 mM MES (pH 5.5, 130 mM NaCl) containing PHEA*-g-*bAPAE (0.5 mg/mL) for 20 min at 37 °C; in the same condition, blank analysis was also performed. After this time 500 µL of Trypsin was added in each well and after 5 min the content of 3 well, treated with the same condition, was reunited in a small centrifuge tube and mixed with 200 µL of Trypan Blue 0.2%. After 1 min the supernatant was removed by centrifugation at 2000 rpm and the residual pellet was washed with 2 mL of DPBS. After washing, the supernatant was removed by centrifugation at 2000 rpm and the pellet in each tube was treated with 250 µL of TRITON X100 1% and plated on a 96-well plate. After 1 h of incubation, the absorbance at 580 nm was read using a Microplate reader (Multiskan Ex, Thermo Labsystems, Vantaa, Finland). All analysis was performed in duplicate.

#### 2.6.3. Cell Viability Assay

Cell viability was assessed by a MTS assay on 16-HBE cells, using a commercially available kit (Cell Titer 96 Aqueous One Solution Cell Proliferation assay, Promega) containing 3-(4,5-dimethylthiazol-2-yl)-5-(3-carboxymethoxyphenyl)-2-(4-sulphophenyl)-2H-tetrazolium (MTS) and phenazine ethosulfate. 16-HBE cells were plated on a 96-well plate at a cell density of 25,000 cells/well in DMEM containing 10% FBS. After 24 h of incubation, the medium was removed and then the cells were incubated with 200 μL per well with an aqueous dispersion (DMEM containing 10% FBS) of each copolymer at concentrations between 2 mg/mL to 0.0625 mg/mL. All polymers dispersions were sterilized by filtration using 220 nm filter. After 24 and 48 h incubation, the polymers dispersions were removed and each plate was washed with sterile DPBS; after this, cells in each well were incubated with with 100 μL of fresh DMEM and 20 μL of a MTS solution and plates were incubated for 2 h at 37 °C. The absorbance at 490 nm was read using a Microplate reader (Multiskan Ex, Thermo Labsystems, Finland). Relative cell viability (percentage) was expressed as (Abs490 treated cells/Abs490control cells) × 100, on the basis of three experiments conducted in multiple of six. Cells incubated with the medium were used as negative control.

### 2.7. Complexation Study

Complexation study were evaluated by gel retardation assay and by measurements of size and potential. Polyplex were formed by adding a volume of the copolymer dispersion at different concentrations to the same volume of siRNA solution at a fixed concentration, in order to obtain different polymer/siRNA weight ratios (R); the mixture was mixed by gently pipetting, followed by 30 min incubation at room temperature, before analysis. For gel retardation assay, siRNA/copolymer polyplexes were formed in nuclease free Hepes buffer 10 mM, at pH7.4, containing glucose 5% (*w/v*). siRNA concentration was 0.1 mg/mL and polymer/siRNA weight ratios (R) were: 0, 1, 2, 2.5, 3, 3.5, 4, and 5. Ten microliters of each sample were then loaded on a 1.5% agarose gel, containing 70 mL ethidium bromide and run at 100 V in trisacetate/EDTA (TAE) buffer at pH 8 for 30 min. The gels were then visualized against an UV trans-illuminator and photographed using a digital camera. For dynamic light scattering studies (DLS), siRNA/copolymer polyplexes were formed in nuclease free Hepes buffer 10 mM, at pH 7.4. siRNA concentration was 0.05 mg/mL and R were 0, 1, 2, 2.5, 3, 4, 5, 7, and 10. DLS measurements were performed on 50 µL of sample at 25 °C with a Malvern Zetasizer NanoZS instrument fitted with a 532 nm laser at a fixed scattering angle of 173°, using the Dispersion Technology Software 7.02. For potential, siRNA/copolymer polyplexes were formed in nuclease free Hepes buffer 10 mM, at pH7.4. siRNA concentration was 0.2 mg/mL and R were 0, 1, 2, 2.5, 3, 4, 5, 7, and 10. Four hundred microliters of each sample was diluted with Hepes buffer until 900 µL befeore measure. potential measurements were performed by aqueous electrophoresis measurements, recorded at 25 °C using the same apparatus for DLS measurement. The potential values (mV) were calculated from the electrophoretic mobility using the Smoluchowski relationship.

### 2.8. Polyplex Stability in Presence of Mucin

#### 2.8.1. Polyanionic Exchange

The stability of polyplexes to polyanionic exchange was determined after polyplexes incubation with mucin dispersion. Polyplexes were prepared as described before in gel retardation assay; the resulting polyplexes (30 µL), were mixed with 5 µL of mucin dispersion (7 mg/mL), in order to have a final mucin concentration of 1 mg/mL, and samples were incubated at room temperature for 2 or 5 h. Gel electrophoresis was then performed as described in complexation study.

#### 2.8.2. Turbidimetric Assay

Measurements of interactions between polyplexes and mucin was determined by turbidimetry. 50 µL of polyplexes, prepared as described in gel retardation assay, were mixed with 50 µL of mucin dispersion at the concentration of 2 mg/mL in Hepes buffer 10 mM pH 7.4. After incubation at 37 °C, the turbidity was measured each 50 min until 6 h approximately. The absorbance at the λ of 500 nm was recorded by Microplate reader (Multiskan Ex, Thermo Labsystems, Finland).

### 2.9. Gene Silencing Assay

The evaluation of the gene silencing capacity was evaluated by ELISA test, using a IL-4 Human ELISA Kit kits from Life Technologies. 16-HBE cells were plated on a 96-well plate at a cell density of 25,000 cells/well in DMEM containing 10% FBS. After 24 h of incubation, the medium was removed and then the cells were incubated with 200 µL of a polyplexes dispersion (for each well 0.01 nmol of siRNA was used) at different R (3, 5, 10) for 48 h; after this time supernatant was removed ed the cells were incubated with 200 µL of LPS 500 ng/mL for 6 h. After this time cells was washed with DPBS and treated following the protocol provided. For this study polyplexes were formed in OPTIMEM medium using a siRNA 0,1 µM. LPS, copolymers, and siRNA solution were sterilized by filtration using 220 nm filter before analysis.

## 3. Results and Discussion

### 3.1. Polymer Synthesis and Characterization

An ideal carrier for achieving the delivery of genetic material into a target tissue must be able, after in vivo administration, to interact with specific cells and to release the genetic material in the cytosol of target cells, overcoming both cellular membrane or the endosomal–lysosomal membrane [7,25]. Synthetic polycations represents in principle valid candidates in this field, thanks to the fact that can be realized with proper structural and functional properties able to confer specific characteristics that a vector of genetic material should have [7]. For this reason, the researchers explored the possibility of producing protonable copolymers with various functionalities in order to confer different properties to a single macromolecule.

Here, a novel polycation derivative of α,β-poly(N-2-hydroxyethyl)-d,L-aspartamide (PHEA) was produced by grafting on the PHEA backbone the 1,2-Bis(3-aminopropylamino)ethane (bAPAE), obtaining the PHEA*-g-*bAPAE graft copolymer. The grafting of bAPAE molecules on PHEA backbone allow to realise a copolymer carrying on the side chains with protonable amines conferring the capability to complex the genetic material by electrostatic interactions.

**Figure 2:**
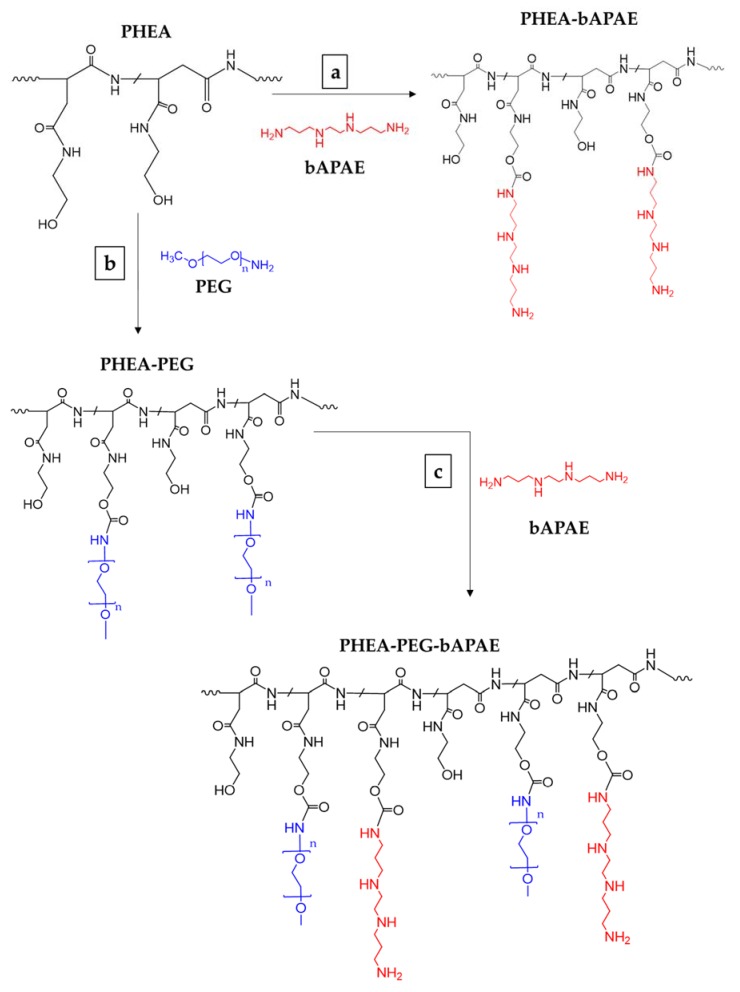
The synthetic route of (**a**) PHEA*-g-*bAPAE and (**b**) PHEA*-g-*PEG and (**c**) PHEA*-g-*PEG*-g-*bAPAE graft copolymers (*n* = 44). Reagents and conditions: a) a-DMF, BNPC, 4 h at 40 °C, 20 h at 25 °C; b) a-DMF, DSC, 4 h at 40 °C, 18 h at 25 °C; b) a-DMF, BNPC, 4 h at 40 °C, 20 h at 25 °C.

The reaction involved the activation of free PHEA hydroxyl groups with bis-nitrophenyl carbonate (BNPC), chosing the stoichiometry of reagents in order to obtain deficiency of BNPC over hydroxyl groups of repeating units (6:10). Using this strategy we obtained a suitable amount of activated groups able to further react with amine functions of bAPAE. However, being a polyamine, a huge excess bAPAE was employed is the second step of the reaction, thus avoiding crosslinking owing to multiple nucleofilic attack of side chains. In these experimental conditions, a derivatization degree in bAPAE (DD_bAPAE_) of PHEA*-g-*bAPAE graft copolymer of about 35 mol% was obtained. The latter was calculated by ^1^H-NMR analysis by using the ratio between the integral of the signals corresponding to 4H of bAPAE (at δ 1.70 and 2.20 ppm), to the integral of the signal corresponding to 2H of PHEA repeating unit (at δ 2.73 ppm); it was confirmend by the colorimetric TNBS assay, that gives a DD% value superimposable to that obtained by ^1^H-NMR analysis [12]. The occurring of the conjugation of 1,2-Bis(3-aminopropylamino)ethane (bAPAE) was demonstrated also by the appearance of signal at about δ 4.1, related to the CH_2_ of the side chain of functionalized PHEA repeat unit (NHCH_2_C**H_2_**OCO-) near to the OCONH bond. The scheme or reaction is reported in Figure 2a.

The obtained copolymer was further characterised by SEC analysis in terms of weight average molecular weight (${\bar{M}}_{w}$) and polydispersity index (${\bar{M}}_{w}/{\bar{M}}_{n})$(for SEC chromatogram, see Figure S1 in Supplementary Materials), and obtained values are reported in Table 2, together with DD_Bapae_ value.

As previously evidenced for other reactions of amine with PHEA, the ${\bar{M}}_{w}$ undergoes a rather drastic reduction, due to the experimental condition to achieve high degree of functionalizations [26]. This fact could be explained with the use of a high amount of amine (four times compared to the RU of PHEA) for carrying out the functionalization reaction of PHEA with bAPAE, that could break some amide bound in the main chain. However authors can not exclude that also the modification of the copolymer conformation respect to that parent polymer can be the reason of the detection of lower molecular weight of copolymers by SEC. However, the use of materials with low ${\bar{M}}_{w}$ is often preferred as polymeric carriers for complexing genetic material [7].

The functionalization of PHEA with bAPAE was done in order to: (a) Make the polymer susceptible to pH changes, that is, modulable in terms of charges to be used for complexing genetic material; and (b) confer it a buffering behavior, that is also a main characteristic request to a genetic vector in order to increase the possibility of giving rise to endosome/lysosome escape, once internalized by cells, due to the so-called proton spoge effect [7].

**Figure 3:**
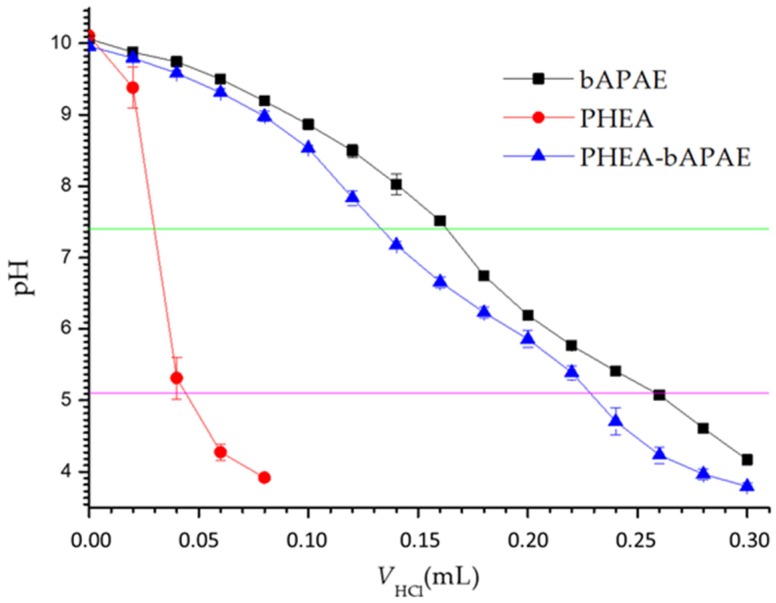
Acid–base titration profiles (pH versus acid volume) of PHEA*-g-*bAPAE (0.2 mg/mL) graft copolymer, PHEA (0.2 mg/mL) and bAPAE moieties (0.047 mg/mL).

The buffering behavior of PHEA*-g-*bAPAE graft copolymer was investigated by an acid−base titration. The titration profile was also obtained for PHEA or bAPAE, in aqueous dispersions, at concentrations present in PHEA*-g-*bAPAE dispersion. Data are reported in Figure 3.

As is evidenced in the graphic, PHEA*-g-*bAPAE copolymer shows a buffering capability in the pH interval from 7.4 (extracellular and cytoplasmatic pH) to 5.1 (endosomal/lysosomal pH), important for endosomal escaping with proton sponge effect [7,27,28]. Moreover, the PHEA*-g-*bAPAE titration profile show a buffering behavior quite superimposable to that obtained by the bAPAE alone. On the contrary, the titration curve of PHEA dispersion showed rapid reduction in pH value, suggesting (as expected) no buffering capacity. This result indicates that the conjugation of the amine confers to the polymeric backbone the buffering capability of the amine itself.

A further potentiometric titration was performed in order to determine the K_a_ constants; titration plot of backward was elaborated using Origin software according to the De Levie method of acid–base equilibria for polyprotic acid and bases, taking into account the activity corrections through the Davies expression (1) [29,30,31,32].

(1) $$\log y=\;-0,5\left(\frac{\sqrt{I}}{1+\sqrt{I}}-0,3I\right)$$

where y is the activity coefficient and I is the ionic strength.

For this titration, the fitting function obtained is the following (2):

(2) $$V_{B}=-\frac{V_{0}\left[C_{0}\left(3\alpha_{3}+2\alpha_{2}+\alpha_{1}\right)+\Delta \right]+V_{A}\left(\Delta -C_{A}\right)}{\Delta +C_{B}}$$

(3) $$\Delta =C_{H^{+}}-\frac{K_{W}}{C_{H^{+}}\cdot y^{2}}+\;C_{B}$$

where V_B_ and C_B_ are respectively the volume and molarity of NaOH for the backword titration, V_0_ is the volume of NaCl using for dissolving PHEA*-g-*bAPAE, C_0_ is the analyte molarity, V_A_ and C_A_ are respectively the volume and molarity of HCl used for the forward titration, while $\alpha_{3},\alpha_{2},\alpha_{1}$ are the protonation degree, K_W_ is the dissociation constant of water and $C_{H^{+}}$ is the H^+^ concentration.

**Figure 4:**
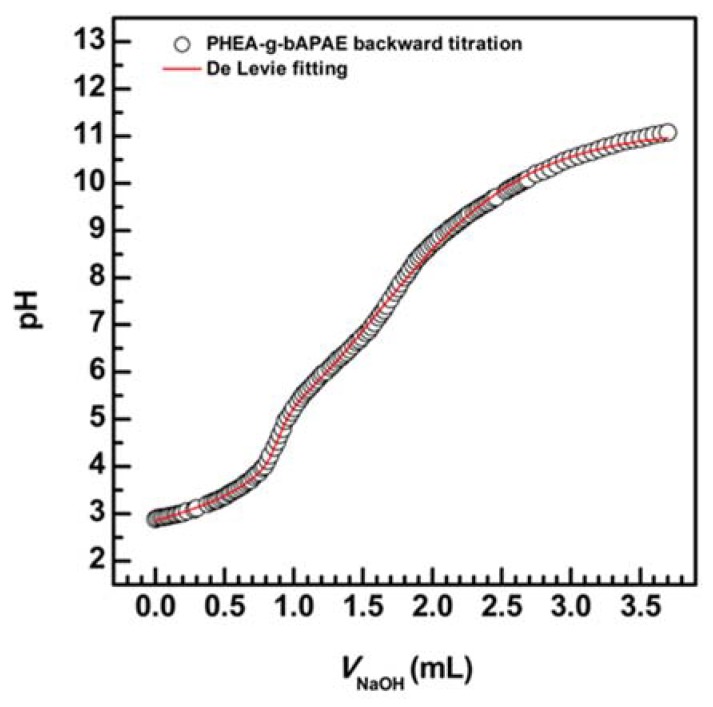
Backward acid–base titration (NaOH volume versus pH) of PHEA*-g-*bAPAE and De Levie fitting curve.

The titration profile and the fitting are reported in Figure 4.

Obtained pK_a1_, pK_a2_, and pK_a3_ values from the curve fitting analysis were found, respectively, equal to 4.8, 7.9, and 10.9.

**Figure 5:**
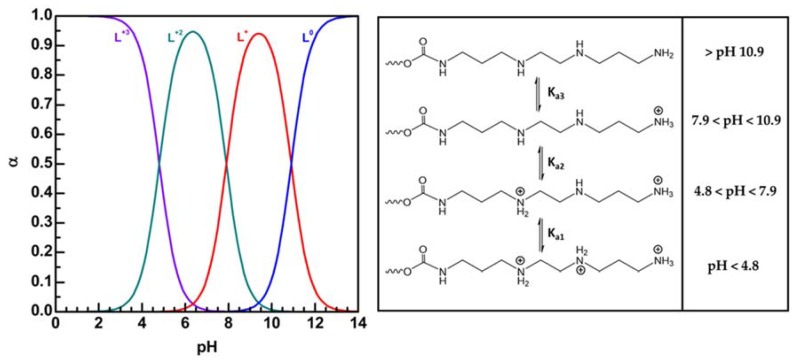
Speciation curves obtained for Plot α versus pH for each α of PHEA-bAPAE PHEA*-g-*bAPAE and protonation state of PHEA-bAPAE PHEA*-g-*bAPAE at different pH values.

As shown in Figure 5, at pH 7.4 the diprotonated species (_L_^+2^) is the mostly present, while, as the pH value of the medium decreases, the amount of the triprotonated species (_L_^+3^) begins to increase, until becoming the mostly present species at a pH value approximately of 4.5. This behavior could determine a different effect of the copolymer on the biological membranes dependin on the pH of the medium, i.e., on cytosolic membranes at pH 7.4 and on endosomal membranes at pH 5 (more or less), demonstrated in our next study.

#### 3.1.1. Membrane Destabilization Study

Once proved its buffering behaviour, the copolymer effect on cell membranes was evaluated in detail as a function of the pH of the medium, i.e., depending on the amount of protonate amines on its structure, by mimicking the cytosol (pH 7.4) and the lysosomal compartment (pH 5).

**Figure 6:**
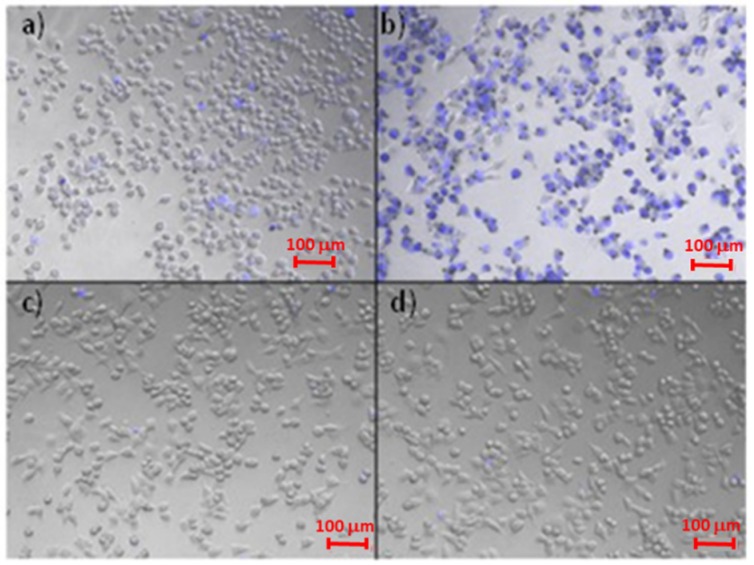
Microscope pictures of 16-HBE treated in different ways: (**a**) cells treated with PHEA*-g-*bAPAE in DPBS pH 7.4. (**b**) cells treated with PHEA*-g-*bAPAE in isotonic MES 20 mM pH 5, (**c**) cells treated with DPBS pH 7.4, (**d**) cells treated with isotonic MES 20 mM pH5.

Therefore, an in vitro study was carried out by using human bronchial cells (16-HBE) as biological membrane model and by incubating these cells in the presence of PHEA*-g-*bAPAE graft copolymer in an aqueous dispersion at two different pH values of the medium: 7.4 and 5. Trypan blue was used as colorant to distinguish healthy cells from those that are dead or in apoptotic state under the microscope, as it is capable of entering cells only when the membranes are destabilized. After incubation for 20 min, cell were treated with Trypan blue and images were recorded and reported in Figure 6.

As shown in figure, considerable cell membrane destabilization occurs only when cell are treated with PHEA*-g-*bAPAE at pH 5, that is in the protonated state, as demonstrated by the fact that trypan blue has free access to all cells. This phenomenon is due to the fact that at pH 5, most of the polymeric species in solution are in the diprotonated state (60%) and in the triprotonated state (40%), while at pH 7.4 most of the polymeric species in solution are in the diprotonated state (75%) and in the monoprotonated state (25%), as showed in Figure 5.

**Figure 7:**
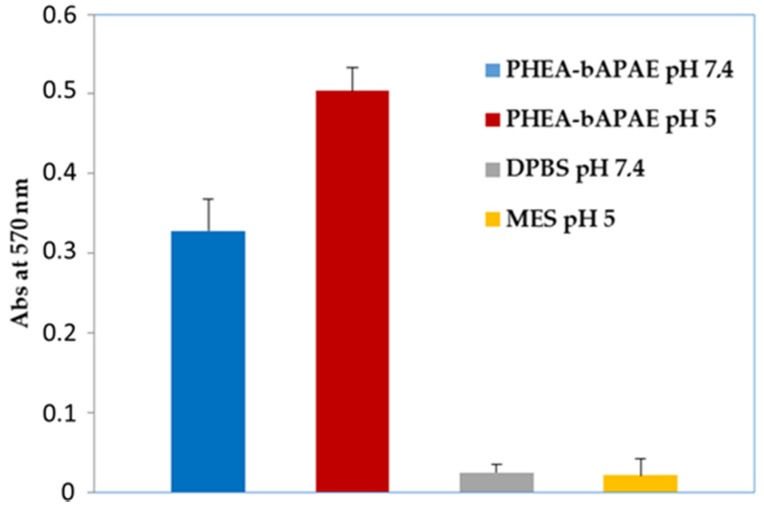
Destabilization assay: absorbance values at 570 nm (trypan blue) in 16-HBE cell dispersion after incubation with PHEA*-g-*bAPAE copolymer at different pH values.

To confirm this result, a quantification of the trypan blue amount was performed by ultraviolet spectrophotometry and obtained values are reported in Figure 7; data shown the maximum absorbance when cells are treated with PHEA*-g-*bAPAE, at pH 5. Even if the quantification does not reflect the same behaviour seen in the microscope images, the results are in any case in agreement with what has been hypothesized, that is the destabilizing effect of the protonated PHEA*-g-*bAPAE graft copolymer on the cellular membrane as a function of pH.

#### 3.1.2. Pegylation

The possibility of using as starting polymer a material that can be easily modified by conjugation with proper molecules is certainly an advantage in cases where it is necessary to take into account the peculiarity of the material to be transported and the barriers to be overcomed after in vivo administration. Here, the PHEA*-g-*bAPAE copolymer was thought to complex siRNA to form polyplexes to be administered by inhalation, then locally to the lung.

Polyethylenglycols (PEG) represent the proper material to influence the properties of the resulting conjugate, thanks to their unique properties such as hydrophilicity and biocompatibility [23]. In most of cases, pegylation of polyplexes provides reduction of toxicity, shielding and stealth properties. However, these improvements are often associated to as a reduction of siRNA condensation, which usually need in using higher amount of polymer [7].

To potentially minimize the tendency to aggregation of polyplexes and interactions with mucus components of lungs, i.e., mucin, PHEA was functionalised with polyethylene glycol (PEG) to obtain PHEA*-g-*PEG graft copolymer. It is well known from previously studies that the mucus-penetrating capability of colloidal carriers through the mucus layer is related to the pegylation degree (DD_PEG_) [23]. PHEA*-g-*PEG*-g-*bAPAE graft copolymer was synthesized by two reaction steps. In the first step, PHEA was left to react with methoxy(polyethylene glycol) amine (CH_3_O-PEG-NH_2_) molecules by using disuccinimidyl carbonate (DSC) as coupling agent; while, in the second step, recovered PHEA*-g-*PEG was left to react with bAPAE by using BNPC as coupling agent. The schematic representation of both steps are reported in Figure 1b,c.

In order to obtain copolymers with a different pegylation degree and to evaluate the effect of the PEG amounts on the interactions with mucin of the resulting polyplexes with siRNA, three different theoretical molar ratio values between CH_3_O-PEG-NH_2_ molecules and PHEA RU (R_1_ = 0.03, 0.075, and 0.12) were used to carry out the reaction, thus obtaining three different PHEA*-g-*PEG graft copolymers with increasing derivatization degrees (DD_PEG_ mol %): 1.9, 2.7, and 4.4 mol %, named respectively PHEA*-g-*PEG(A), PHEA*-g-*PEG(B) and PHEA*-g-*PEG(C).

The DD values were calculated by ^1^H-NMR analysis as the ratio between the integral of the signals corresponding to protons on PEG (at δ 3.60 ppm), to the integral of those corresponding to 2H of PHEA repeating unit (at δ 3.24 ppm).

All the obtained PHEA*-g-*PEG copolymers were characterised in terms of ${\bar{M}}_{w}$ and ${\bar{M}}_{w}/{\bar{M}}_{n}$ (for SEC chromatograms, see Figure S1 in Supplementary Materials), and obtained values are reported in Table 2, together with chemical composition expressed as DD_PEG_. The increase in the copolymer ${\bar{M}}_{w}$ is in accordance with the theoretical value calculated considering thr starting PHEA ${\bar{M}}_{w}$ value and the DD in PEG of each copolymer.

**Figure 8:**
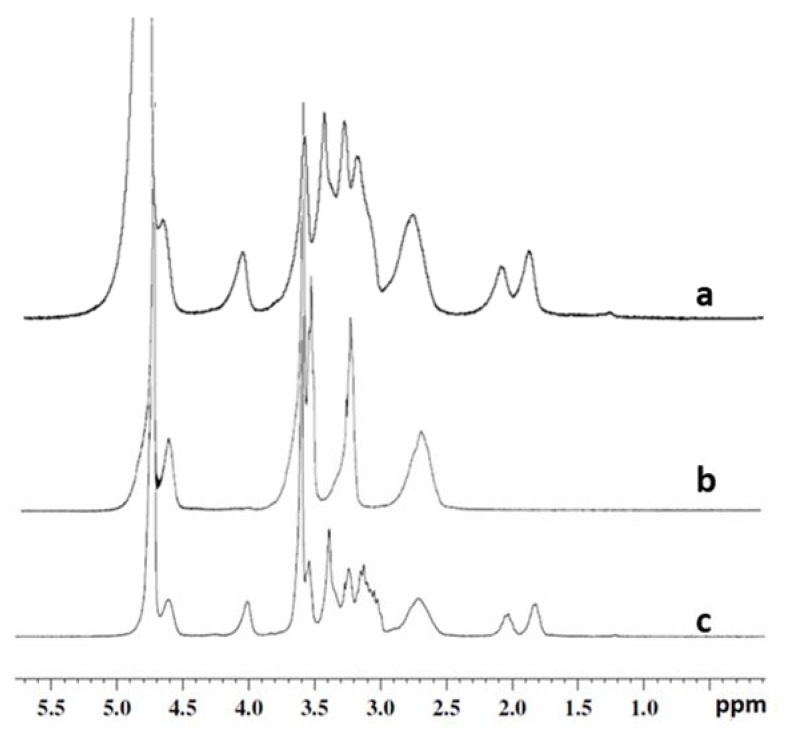
The typical ^1^H-NMR spectrum of **a**) PHEA*-g-*bAPAE (D_2_O at pD < 5), **b**) PHEA*-g-*PEG(C) (D_2_O), and **c**) PHEA*-g-*PEG(C)*-g-*bAPAE (D_2_O at pD < 5).

In the second step, PHEA or PHEA*-g-*PEG(A)-(C) graft copolymers were further functionalized with bAPAE by using BNPC, obtaining a DD_bAPAE_ of each PHEA*-g-*PEG*-g-*bAPAE graft copolymers of about 35 mol%. The latter was calculated by ^1^H-NMR analysis as reported before for PHEA*-g-*bAPAE graft copolymer. The typical ^1^H-NMR spectrum of a PHEA*-g-*PEG*-g-*bAPAE graft copolymer is reported in Figure 8.

The obtained DD mol% was also confirmend by the colorimetric TNBS assay, that gives a DD% value superimposable to that obtained by ^1^H-NMR analysis. All the obtained copolymers were characterised in terms of ${\bar{M}}_{w}$ and ${\bar{M}}_{w}/{\bar{M}}_{n}$ (for SEC chromatogram, see Figure S1 in Supplementary Materials), and obtained values are reported in Table 2, together with chemical composition espressed as DD_PEG_ and DD_bAPAE_ mol%. In this case, the reduction in the ${\bar{M}}_{w}$ of each PHEA*-g-*PEG starting copolymer is in agreement with what described before for PHEA following the reaction with the amine bAPAE. Moreover, the differences between experimental and theoretical ${\bar{M}}_{w}$ values of PHEA*-g-*PEG*-g-*bAPAE graft copolymers could be attributed to conformational modifications of obtained copolymers in the aqueous medium depending on the amount of linked PEG.

#### 3.1.3. Cell Viability Assay

**Figure 9:**
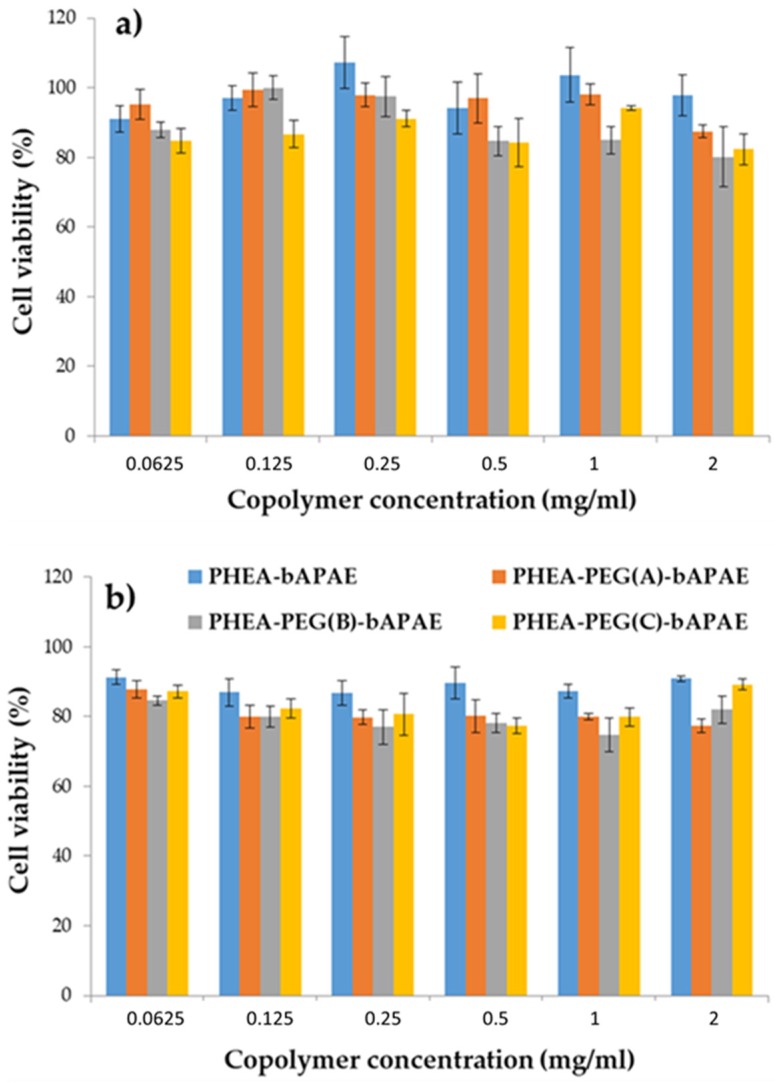
16-HBE viability assay after (**a**) 24 and (**b**) 48 h of incubation with all copolymers.

Considering the potential application of the PHEA*-g-*bAPAE and/or PHEA*-g-*PEG*-g-*bAPAE graft copolymers as a starting material to realise a formulation for pulmonary administration of siRNA, cytocompatibility of all copolymers was evaluated by the MTS assay on 16-HBE cells at different concentrations, after 24 and 48 incubation. Results are shown in Figure 9a,b.

As can be seen, also after 48 h incubation, all copolymers showed a good cytocompatibility at all tested concentrations, showing a cell viability higher than 80% compared to the control experiment, where cells are incubated only with DMEM medium.

### 3.2. Complexation Studies and Characterization of Obtained Polyplexes

The obtained copolymers, PHEA*-g-*bAPAE, PHEA*-g-*PEG(A)*-g-*bAPAE, PHEA*-g-*PEG(B)*-g-*bAPAE, and PHEA*-g-*PEG(C)*-g-*bAPAE, possess in their chemical structure an equal amount of amine (about 35 mol%) and increasing PEG chains (about 0, 1.9, 2.7, and 4.4 mol%) linked on the PHEA backbone, that could reduce the capability to the polyplexes to interact with mucus components but at the same time, the capability to electrostatically interact with siRNA and to be internalized by cells. For this reason, all four were used for the subsequent evaluation of the siRNA complexing capacity. As therapeutic siRNA, a siRNA able to reduce the expression of STAT6 was chosen, that regulate the production of Th2 cytokines and effector functions mediated by Th2 cytokines [4], and that which seems to have a major role in the mechanism that initiates an asthmatic attack.

**Figure 10:**
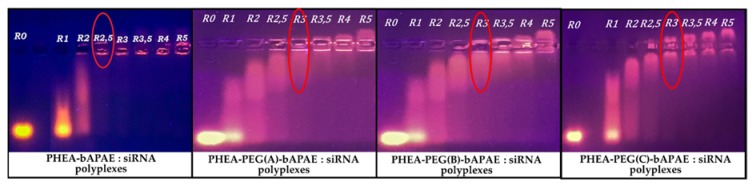
Agarose gel electrophoresis of polyplexes obtained in HEPES 10 mM at various graft copolymer to siRNA weight ratios (R) ranging between 1 and 5.

To understand if each synthetized copolymer can electrostatically bind negatively charged siRNA molecules, complexation studies were performed. To do this, a mixing of two equal volumes of two dispersions, on containing a fixed concentration of siRNA and the other one containing increasing concentration of each copolymer was done, in order to obtain different polymer/siRNA weight ratios (R) ranging between 1 and 5. To evaluate the formation of stable complexes, an electrophoresis analysis on agarose gel was performed; results are reported in Figure 10.

As can be seen in Figure 10, each PHEA*-g-*PEG*-g-*bAPAE copolymer was able to retard the electrophoresis run of siRNA molecules starting from a polymer/siRNA weight ratio of 3; on the other hand, PHEA*-g-*bAPAE was able to retard the electrophoresis run of siRNA molecules starting from a polymer/siRNA weight ratio of 2,5. This difference probably is due to the fact that the same amounts of PHEA*-g-*bAPAE and PHEA*-g-*PEG*-g-*bAPAE copolymers contain a different amounts of amine groups, that are higher in PHEA*-g-*bAPAE due to the absence of PEG chains in the polymeric backbone. In effect, if the N/P ratio is considered, it means that the unpegylated copolymer stop the siRNA run at N/P equal to 3.5, while for PHEA*-g-*PEG(A)*-g-*bAPAE, PHEA*-g-*PEG(B)*-g-*bAPAE PHEA*-g-*PEG(C)*-g-*bAPAE graft copolymers correspond to a N/P ratio of 3.5, 3.4, and 3, respectively. This result means that the pegylated copolymers were able to retard the electrophoresis run of siRNA molecules at lower N/P ratios than PHEA*-g-*bAPAE graft copolymer, and that increasing the PEG amount improves this behaviour. In all cases, the R need to stop the mobility of siRNA is quite low if compared to other synthetic copolymers proposed as non viral vectors for siRNA terapy, as reported elsewhere [7].

**Figure 11:**
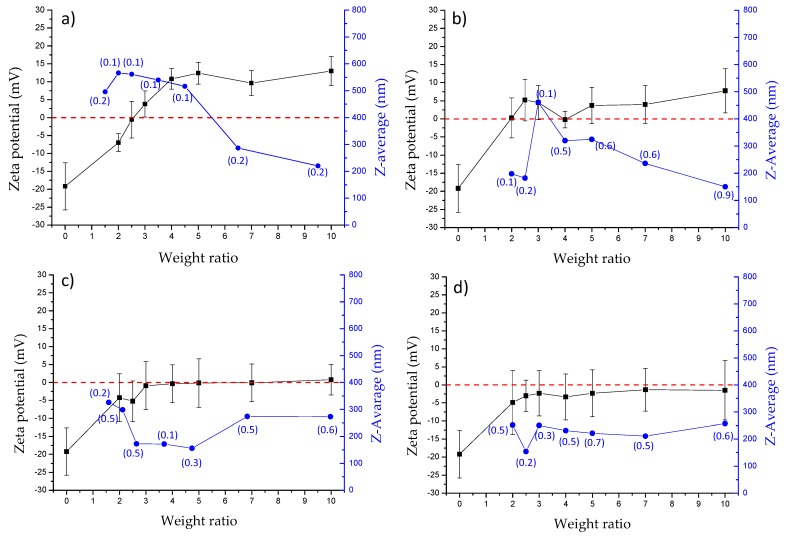
Mean size (blue line), PDI (value enclosed in brakets) and potential (black line) of polyplexes formed by siRNA and: (**a**) PHEA*-g-*bAPAE; (**b**) PHEA*-g-*PEG(A)*-g-*bAPAE; (**c**) PHEA*-g-*PEG(B)*-g-*bAPAE; (**d**) PHEA*-g-*PEG(C)*-g-*bAPAE graft copolymers, at weight ratios (R) ranging between 0 and 10 (data are reported as means SD, *n* = 3).

In order to confirm these results, the mean size, PDI and potential values of obtained polyplexes at different R values (ranging between 1 and 10) were measured and obtained data reported in Figure 11 for each synthesized copolymer.

As expected, for PHEA*-g-*bAPAE based polyplexes, a negative potential is recorded for the lowest weight ratio; the potential increases then, up to the point of reversing itself, as a higher quantity of copolymer is mixed with siRNA. Regarding to size profile, higher dimensions of the polyplexes are measured as they present a potential near neutrality, which decrease when the potential of the polyplex increases; this phenomenon is due to the fact that near a zero potential, phenomena of aggregation between polyplexes occur, while when the potential deviates from neutrality, the phenomena of repulsion cause a reduction of this aggregation. For polyplexes obtained with pegylated copolymers, a slightly different behaviour is recorded. First, regarding the potential, there are no high potential values even for the greatest weight ratios, but a stasis is observed in the region of neutrality; this phenomenon gradually increases with the increase in the amount of PEG present in the polymeric backbone. This behaviour is probably due to the ability of the PEG chains to form a shell that shields the surface charges. But at the same time, this shell causes a reduction of the phenomena of aggregation; in fact, there are no large and quite static dimensions, especially for the complexes obtained by PHEA*-g-*PEG(C)*-g-*bAPAE, where the PEG amount is higher. Thus, the presence of PEG in the copolymer allows to obtain polyplexes with siRNA that does not aggregate while maintaining a charge close to neutrality.

### 3.3. Interaction Studies of Polyplexes with Mucin

Being the copolymer/siRNA complexes in view to be administered by the inhalation route, it was necessary to assess whether these polyplexes interact with the mucin and if the presence of PEG in the copolymer structure can effectively influence these interactions, given that the mucus layer represents the main barrier that the inhaled particles must overcome.

**Figure 12:**
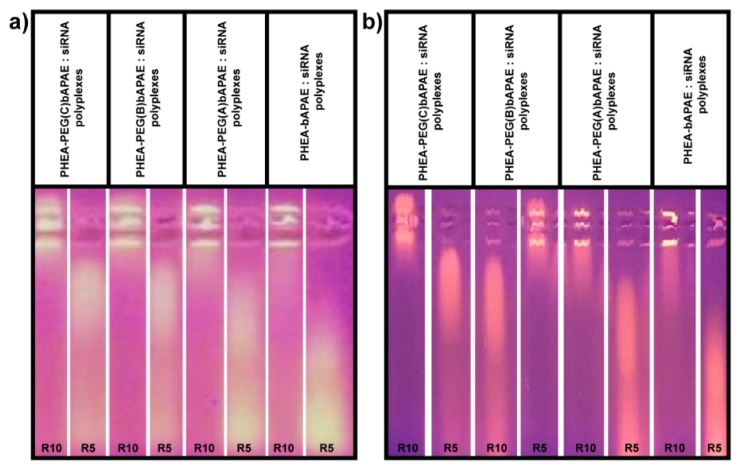
Gel electrophoresis in the presence of mucin, after (**a**) 2 h and (**b**) 5 h of incubation, at R = 5 and 10.

The first study was carried out considering that, given the polyanionic nature of the mucin (due to the presence of sialic acid residues), a polyanionic exchange between siRNA and mucin may occur. Thus, a study was carried out by evaluating the electrophoretic mobility of siRNA in the polyplexes in the presence of mucin in the dispersion medium. In particular, the study was carried out in the presence of mucin at a concentration of 1 mg/mL, for 2 and 5 h. Obtained images for R = 5 and 10 are reported in Figure 12.

As showed in Figure 12, all the polyplexes give a polyanionic exchange between siRNA and mucin, so that a higher weight ratio (R = 10) needs to stop the electrophoretic run of siRNA respect to that request in the absence of mucin, as reported in Figure 10.

**Figure 13:**
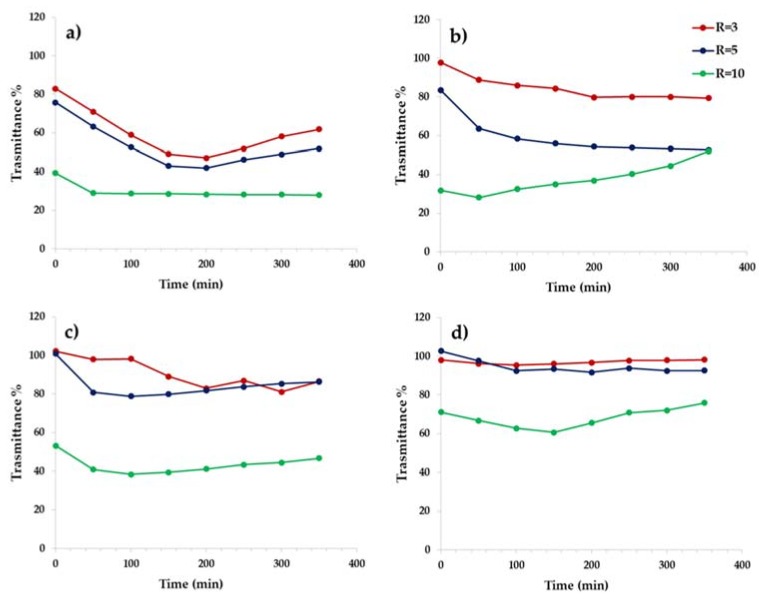
Turbidimetric analysis. Trasmittance at 500 nm of a dispersion containing mucin in the presence of polyplexes between siRNA and: (**a**) PHEA*-g-*bAPAE; (**b**) PHEA*-g-*PEG(A)*-g-*bAPAE; (**c**) PHEA*-g-*PEG(B)*-g-*bAPAE; (**d**) PHEA*-g-*PEG(C)*-g-*bAPAE graft copolymers, at R equal to 3, 5, and 10.

Therefore, the mucin competes with the siRNA for the electrostatic interactions with the copolymer, so that a higher amount of copolymer is request to form polyplexes. In order to obtain more informations regarding the specific interaction of each copolymer forming the polyplex with mucin, a turbidimetric assay was carried out at three different R values (3, 5, and 10) as a function of incubation time, considering that if polyplexes-mucin interactions occur, it involves a reduction of transmittance of the dispersion. Data are reported in Figure 13a–d, as trasmittance % as a function of incubation time.

As can be seen, polyplexes obtained with PHEA*-g-*bAPAE shown a muco-adhesive behaviour, especially as the weight ratio value R increases. The dependence from R is also showed by the polyplexes obtained with the pegylated copolymers. On the other hand, for the pegylated polyplexes, the interaction capability with mucin decreases as the amount of PEG in the polymeric backbone increases to all selected R values. The polyplexes obtained with PHEA*-g-*PEG(C)*-g-*bAPAE seems to be not susceptible to the presence of mucin, showing high trasmittance values at all the incubation times also at higher concentration (corresponding to R = 10). The latter also represents the minimum weight ratio to avoid the polyanionic exchange of complexed siRNA with mucin, thus showing adequate behavior to be used as an effective vector for siRNA.

### 3.4. Gene Silencing Assay

**Figure 14:**
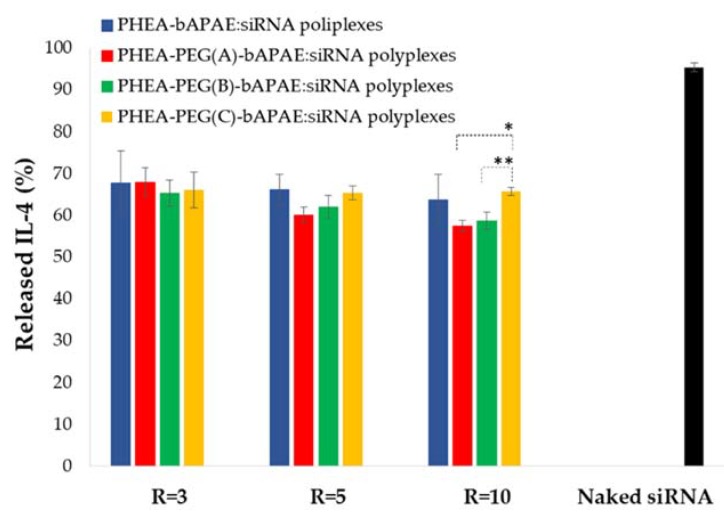
IL-4 (%) released by 16-HBE cells after incubation with polyplexes or with naked siRNA for 48 hrs, compared to positive control. (*p < 0.01; **p < 0.001).

Once demonstrated that these polyplexes are stable the capability to act as an effective carrier for siRNA and to allow the cellular internalization was evaluated. In particular, the gene silencing capacity of obtained polyplexes was evaluated by in vitro ELISA test upon 16-HBE cells. Cells were treated with: a) Naked siRNA and b) polyplexes obtained with each copolymer and at three different R, and then exposed to an inflammatory agent, such as LPS. Relative IL-4 production (percentage) was expressed as (Abs treated cells/Abs positive control cells) × 100. Obtained values are reported in Figure 14.

Obtained results show that cell incubated with naked siRNA produce an IL amount non significantly different from that obtained with control cells. When cells are incubated in the presence of polyplexes, there is a reduction in the expression of IL-4 in all cases, even if not in a massive way. However, this experiment showed that polymeric carrier has a fundamental role in cellular transport and uptake of siRNA. Another important result is given by the fact that presence of PEG does not seem to hinder the cellular uptake of polyplexes, as found for other pegylated non viral polymeric vectors for siRNA [7]. However, at R = 10, among the pegylated polyplexes, the capability to allow the intracellular delivery of siRNA is inversally related to the PEG amount in a significant way, while at the other R no significant differences are observed.

## 4. Conclusions

The discovery of new targets for the treatment of pathologies has gone hand in hand with the need for new carriers able to allow an adequate release to the site of action and to obtain the maximum effectiveness on the treated cells. Gene therapy using non-viral vectors is very popular today, especially because it is safer than using viral vectors, although less efficient. Therefore, the realization of synthetic polymeric carriers has allowed us to design a polymer and its realization by adding perfectly functional components to its structure.

In this work, a protonable copolymer was designed for the production of polyplexes with a siRNA for the treatment of asthma. It was obtained by using PHEA as starting polymer, that is highly functionalisable, and as molecule to be conjugated in a side chain to complex siRNA, we have chosen a oligoamin bAPAE. The latter was bonded in a suitable quantity by means of a easy to reproduce reaction. Since it was thought to administer the polyplex directly to the lung, the carrier has been appropriately functionalized with variable amounts of PEG, since it is already known that the latter molecule is able to modulate the hydrophilicity, shield the charge of the material, and thus confer the ability to penetrate through a mucus layer.

All the obtained copolymers, pegylated or not, were able to complex the selected siRNA at quite low values of weight ratios, giving nanosized complexes with a tendence to the aggregation invertially proportional to the pegylation degree. These polyplexes seem to be destabilized by mucin, that gives polyanionic exchange with siRNA by direct electrostatic interactions of each copolymer with mucin. However, these interactions decrease as a function of the amount of linked PEG, resulting very low for the highest pegylated polyplexes based on the PHEA*-g-*PEG(C)*-g-*bAPAE copolymer. Moreover, the complexation of siRNA with all the synthesized copolymers significantly increases the ability of complexed siRNA to be internalized by the 16 HBE cells. Therefore, the PHEA*-g-*PEG(C)*-g-*bAPAE copolymer possesses interesting potential as a pulmonary siRNA carrier, being not interacting with mucins and able to give neutral polyplexes with quite static dimensions in a wide range of weight ratio.

Furthermore, the performance of PHEA*-g-*PEG(C)*-g-*bAPAE copolymer, as siRNA delivery system, is going to be improved by grafting on the copolymer structure other functional moieties such as cell penetrating peptides, able to improve cell internalization of the siRNA/copolymer polyplexes and/or cross-linking agents able to increase polyplexe stability in biological environment.

## Figures and Tables

**Table 1:** Molar ratio and mL of poly(ethyleneglycole) (PEG) solution (50 mg/mL) used for synthesis.

Copolymers	R_3_	R_4_	R_5_	PEG Solution (mL)	PEG Weight Amount (mg)
PHEA*-g-*PEG(A)	0.03	0.04	1	4	200
PHEA*-g-*PEG(B)	0.075	0.1	1	9.5	475
PHEA*-g-*PEG(C)	0.12	0.16	1	15	750
R_3_ = (mmol of aminoPEG/mmol of functionalizable RU on PHEA) R_4_ = (mmol of DSC/mmol of functionalizable RU on PHEA) R_5_ = (mmol of TEA/mmol of DSC)					

**Table 2:** Weight-average molecular weight (${\bar{M}}_{w}$), polydispersity index (${\bar{M}}_{w}/{\bar{M}}_{n})$, and chemical composition of obtained copolymers.

Copolymers	Molecular Weight		Degree of Derivatization (DD)	
	${\bar{M}}_{w}\;(g/\mathrm{mol})$	${\bar{M}}_{w}/{\bar{M}}_{n}$	DD_PEG_	DD_bAPAE_
PHEA	67500	1.24	---	---
PHEA*-g-*PEG(A)	82,410	1.2	1.9	---
PHEA*-g-*PEG(B)	95,360	1.3	2.7	---
PHEA*-g-*PEG(C)	110,800	1.3	4.4	---
PHEA*-g-*bAPAE	20,921	1.41	---	34
PHEA*-g-*PEG(A)*-g-*bAPAE	25,400	1.32	1.9	35
PHEA*-g-*PEG(B)*-g-*bAPAE	32,100	1.35	2.7	36
PHEA*-g-*PEG(C)*-g-*bAPAE	34,700	1.41	4.4	33

## Supplementary Materials

The following are available online at https://www.mdpi.com/1999-4923/12/2/89/s1, Figure S1: SEC chromatograms of: a) PHEA-g-bAPAE (black), PHEA-g-PEG(A)-g-bAPAE (blue), PHEA-g-PEG(B)-g-bAPAE (red), PHEA-g-PEG(c)-g-bAPAE (green); b) PHEA-g-PEG(A) (black), PHEA-g-PEG(B) (red), PHEA-g-PEG(C) (green).
