# Supplementary material for: Design of New Polyaspartamide Copolymers for siRNA Delivery in Antiasthmatic Therapy
Source: Pharmaceutics. 2020 Jan 22;12(2):89. doi: 10.3390/pharmaceutics12020089 (PMC7076449; doi:10.3390/pharmaceutics12020089)
Supplement: Supplementary file 1 [file pharmaceutics-12-00089-s001.pdf]

pharmaceutics

MDPI

Supplementary Materials: Design of New Polyaspartamide Copolymers for siRNA Delivery in Antiasthmatic Therapy

Emanuela Fabiola Craparo, Salvatore Emanuele Drago, Nicolò Mauro, Gaetano Giammona and Gennara Cavallaro

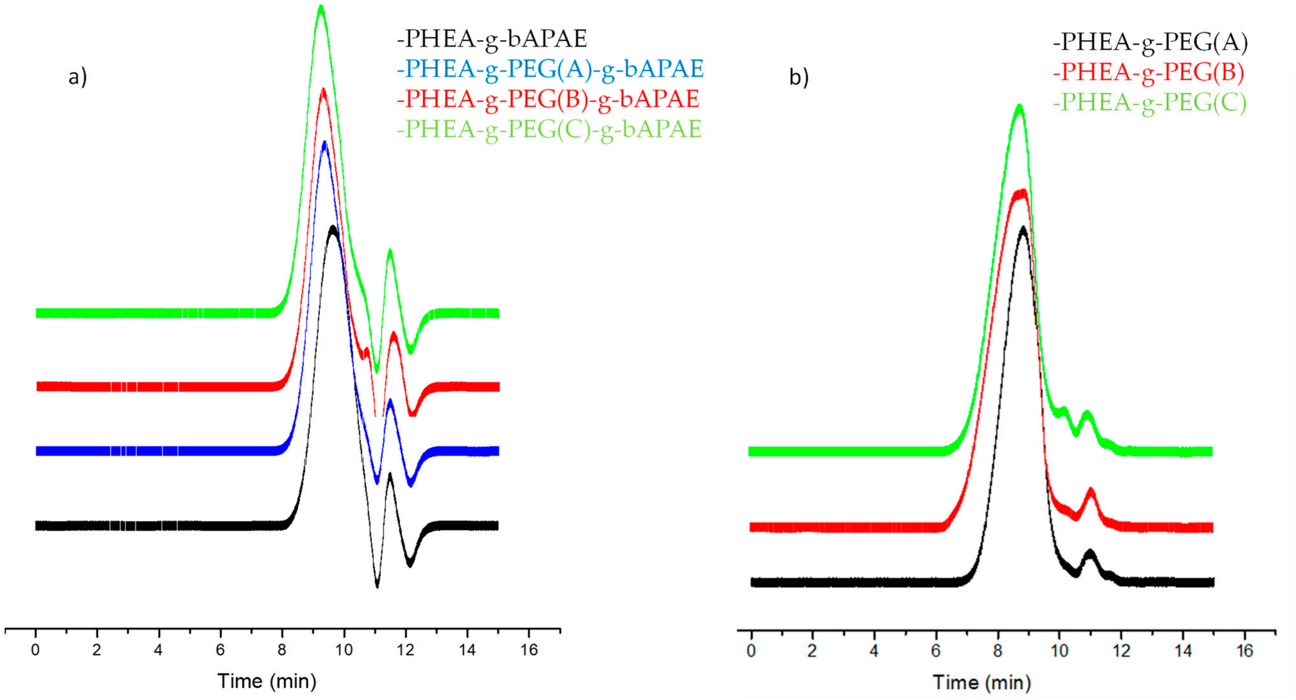

Figure S1. SEC chromatograms of: a) PHEA-g-bAPAE (black), PHEA-g-PEG(A)-g-bAPAE (blue), PHEA-g-PEG(B)-g-bAPAE (red), PHEA-g-PEG(c)-g-bAPAE (green); b) PHEA-g-PEG(A) (black), PHEA-g-PEG(B) (red), PHEA-g-PEG(C) (green).
